# Fixing fractures of the ulnar styloid in distal radius fractures

**DOI:** 10.1186/1753-6561-9-S3-A43

**Published:** 2015-05-19

**Authors:** Marcos Sanmartin Fernandez

**Affiliations:** 1 Department of Orthopaedics and Traumatology, Hospital Povisa, Vigo, 36211, Spain

## 

The majority of distal radius fractures are associated with an ulnar styloid fracture [1-3]. There is solid evidence in the literature that this association may ultimately affect the clinical outcome causing instability, disability, and pain in the distal radioulnar joint (DRUJ) [2,4-7]. The main reasons for the worse outcome are: (1) DRUJ instability, (2) impingement with the triquetrum, and (3) Extensor Carpi Ulnaris (ECU) tenosynovitis.

Because of its close association with the Triangular Fibrocartilage Complex (TFCC), fractures of the ulnar styloid might indicate possible TFCC disruption with subsequent DRUJ instability [4,8,9]. Studies on the clinical relevance of ulnar styloid fractures have produced conflicting results. However, when considering size and displacement of the fracture, May et al [2] found that, after distal radius fracture stabilization, a fracture through the base of the ulnar styloid that involves most part of the TFCC attachment and stays in a deviated more radial position (2 mm or more) indicates DRUJ instability. In an ulnar styloid fracture model, Shaw et al [4] found that K-wire fixation of the ulnar styloid fracture restored stability to the DRUJ regardless the integrity of other supporting structures.

Bone fragments resulting from non-union could also provoke irritation and pain [5,10] due to impingement with the triquetrum [11]. Twenty-six percent of ulnar styloid fractures develop non-union [12]. From those, just a few becomes symptomatic [5]. If the DRUJ is stable, simple removal of the fragment usually solves the problem [5,11]. Extensor carpi ulnaris tendon impingement caused by malalignment of a fibrous nonunion may also be responsible for a poor clinical outcome. [13,14]. This condition is rarely found after a distal radius fracture and it is successfully treated with excision of the bony prominence and reconstruction of the floor of the sixth compartment. Hence, fixation of a small sized ulnar styloid in the acute setting of a distal radius fracture is probably unnecessary.

As the results of treatment of chronic instability of DRUJ following a distal radius fracture are more unpredictable [4], it is advisable to take all measures in the acute setting to prevent this complication. Therefore, fixation of the ulnar styloid is indicated when there are large sized fragments that persist deviated after stabilization of the radius or when the distal radioulnar joint is grossly unstable. Immobilization with a sugar tong splint for four weeks or until proper healing is necessary when the surgeon chooses not to fix a reduced ulnar styloid large fragment that is acceptably reduced.
